# The Reversed Feto-Maternal Bile Acid Gradient in Intrahepatic Cholestasis of Pregnancy Is Corrected by Ursodeoxycholic Acid

**DOI:** 10.1371/journal.pone.0083828

**Published:** 2014-01-08

**Authors:** Victoria Geenes, Anita Lövgren-Sandblom, Lisbet Benthin, Dominic Lawrance, Jenny Chambers, Vinita Gurung, Jim Thornton, Lucy Chappell, Erum Khan, Peter Dixon, Hanns-Ulrich Marschall, Catherine Williamson

**Affiliations:** 1 Institute of Reproductive and Developmental Biology, Imperial College London, London, United Kingdom; 2 Departments of Clinical Chemistry and Medicine, Karolinska Institutet, Stockholm, Sweden; 3 School of Human Development, University of Nottingham, Nottingham, United Kingdom; 4 Women's Health Academic Centre, King's College London and King's Health Partners, London, United Kingdom; 5 Sahlgrenska Academy, University of Gothenburg, Gothenburg, Sweden; Clermont Université, France

## Abstract

Intrahepatic cholestasis of pregnancy (ICP) is a pregnancy-specific liver disorder associated with an increased risk of adverse fetal outcomes. It is characterised by raised maternal serum bile acids, which are believed to cause the adverse outcomes. ICP is commonly treated with ursodeoxycholic acid (UDCA). This study aimed to determine the fetal and maternal bile acid profiles in normal and ICP pregnancies, and to examine the effect of UDCA treatment. Matched maternal and umbilical cord serum samples were collected from untreated ICP (n = 18), UDCA-treated ICP (n = 46) and uncomplicated pregnancy (n = 15) cases at the time of delivery. Nineteen individual bile acids were measured using HPLC-MS/MS. Maternal and fetal serum bile acids are significantly raised in ICP compared with normal pregnancy (p = <0.0001 and <0.05, respectively), predominantly due to increased levels of conjugated cholic and chenodeoxycholic acid. There are no differences between the umbilical cord artery and cord vein levels of the major bile acid species. The feto-maternal gradient of bile acids is reversed in ICP. Treatment with UDCA significantly reduces serum bile acids in the maternal compartment (p = <0.0001), thereby reducing the feto-maternal transplacental gradient. UDCA-treatment does not cause a clinically important increase in lithocholic acid (LCA) concentrations. ICP is associated with significant quantitative and qualitative changes in the maternal and fetal bile acid pools. Treatment with UDCA reduces the level of bile acids in both compartments and reverses the qualitative changes. We have not found evidence to support the suggestion that UDCA treatment increases fetal LCA concentrations to deleterious levels.

## Introduction

Intrahepatic cholestasis of pregnancy (ICP) is characterised by maternal pruritus and deranged liver function. It typically presents in the third trimester, and is associated with an increased risk of adverse fetal outcomes, including fetal distress, spontaneous pre-term labour and intrauterine death. Serum bile acids are the most sensitive and specific biochemical marker of cholestasis in pregnancy [1]. The aetiology of the fetal complications is thought to relate to the deleterious effects of bile acids crossing the placenta and accumulating in the fetal compartment. Transplacental transfer of bile acids has been demonstrated in the rodent and sheep models of ICP [2]–[4], but data demonstrating direct transfer of bile acids across intact human placentas are lacking and data relating to specific molecular pathways for the transport of bile acids in placental tissue are limited [5]–[9]. However, indirect evidence for the transfer of bile acids from mother to fetus comes from studies involving the concurrent measurement of bile acids in matched maternal and umbilical cord serum [10], [11]. These studies identified transplacental gradients that facilitate clearance of these toxic compounds in normal pregnancies, but are reversed in cholestatic pregnancies [12], [13], thereby contributing to the accumulation of bile acids in the fetal compartment.

Several studies have examined the serum bile acid profiles in small numbers of ICP cases. These have demonstrated that the predominant bile acid is cholic acid (CA) with a relative reduction in the proportion of chenodeoxycholic acid (CDCA). This is in contrast to a normal pregnancy where CDCA is present at similar, or slightly higher levels than CA. The level of deoxycholic acid (DCA) has been reported to be either decreased or increased, although both changes occur to a lesser extent than those affecting the primary bile acids [14]–[20]. There are also qualitative changes in the bile acid pool in ICP, with a shift towards taurine conjugates and a consequent reduction in the glycine: taurine ratio [14].

ICP is commonly treated with ursodeoxycholic acid (UDCA), a tertiary bile acid present in small amounts (1–3%) in normal human serum. UDCA treatment is reported to reduce total serum bile acid levels in the maternal and fetal compartments [1], [21], [22], as well as normalising the maternal CA:CDCA and glycine:taurine ratios [14]. Furthermore, several experimental models suggest that UDCA may have a direct protective effect on the fetal compartment [23], [24]. Lithocholic acid (LCA) is a monohydroxy bile acid produced from the metabolism of CDCA in the gut. It has also been reported that UDCA can be converted to LCA, which may be of clinical importance as LCA is more hydrophobic than other bile acids and therefore more toxic [25].

The aim of this study was to determine the profiles of 15 individual bile acid species in matched maternal and fetal serum samples from normal and ICP pregnancies and to investigate the effect of treatment with UDCA, with particular reference to the levels of LCA.

## Patients and Methods

### Patient Population and Sample Collection

Maternal and umbilical cord blood samples were collected from women with ICP (n = 64) and uncomplicated pregnancy (n = 15) receiving antenatal care at Queen Charlottes and Chelsea Hospital, London, Nottingham City Hospital and Queen's Medical Centre, Nottingham. ICP was diagnosed as previously described [26]. All women received antenatal care, including treatment for ICP, in accordance to local hospital policies. 46 of the women with ICP were treated with UDCA, 18 were untreated. Control subjects were women with an uncomplicated pregnancy. Specifically they had no history in the current or any previous pregnancy of pruritus or deranged liver function. Where possible separate blood samples were collected from the umbilical cord artery and vein (5 from UDCA-treated ICP pregnancies, 7 from untreated ICP pregnancies and 13 from controls). All other umbilical cord samples contained mixed arterial and venous umbilical cord blood.

The clinical characteristics of the ICP women are shown in Table 1.

**Table 1:** Biochemical features of the intrahepatic cholestasis of pregnancy (ICP) cases.

	N	Gestation at Diagnosis (weeks + days)	Gestation at Delivery (weeks + days)	Duration of Treatment (weeks + days)	Peak BA* (µmol/L)	Peak AST* (IU/L)	Peak ALT* (IU/L)
**Untreated**	18	34^+5^ (30^+2^–36^+0^)	37^+3^ (36^+3^–38^+1^)	n/a	29 (22–42)	68 (38.5–146.5)	133 (77–185)
**Treated**	46	35^+0^ (29^+4^–37^+0^)	37^+2^ (36^+0^–37^+5^)	4^+6^ (3^+4^–6^+6^)	44 (21–79)	71 (53.5–146.5)	144 (93.5–262)

Serum biochemistry measurements represent the peak concentrations measured during the pregnancy.

BA – bile acids (normal range <14 µmol/L); AST – aspartate transaminase (normal range 5–31 IU/L); ALT – alanine transaminase (normal range 5–31 IU/L); n/a – not applicable.

Values represent median and interquartile ranges.

### Ethics Statement

This study was approved by Hammersmith Hospital Research Ethics Committee and written informed consent was obtained from all participants (REC reference numbers. 97/5197 and 08/H0707/21).

### Sample Preparation

Blood samples were collected in plain vacutainers and centrifuged at 3500 rpm for 10 minutes. The serum was stored at −80°C until use.

250 mL of serum was added to 800 ng of a combination of deuterium labelled bile acids as internal standards (see below) in 40 mL methanol and 800 mL acetonitril. The mixture was centrifuged and the supernatant collected and blown to dryness under a stream of nitrogen. The pellets were redissolved in 75 mL methanol and transferred to autosampler vials for analysis.

### HPLC-MS/MS analysis of serum bile acids

Deuterium labelled unconjugated and glycine- or taurine conjugated bile acids used as internal standards for quantification were obtained from QMX Laboratories, Thaxted, UK. Unconjugated and glycine or taurine conjugated bile acids used as reference compounds were obtained from Sigma-Aldrich (Gillingham, UK).

Serum bile acids were analysed using high performance liquid chromatography- tandem mass spectrometry (HPLC-MS/MS) on a Waters LC-MS/MS Quattro Micro, equipped with a C18 reverse- phase column and operated in ESI negative mode. Quantification was achieved by comparison of peak height of molecular anions or negative daughter ions (for unconjugated and conjugated bile acids, respectively) to the peak height of the deuterated internal standards. The detection limit was approximately 0.010 µmol/L, linearity was confirmed between 0.075 and 200 µmol/L.

### Statistical Analysis

Mann-Whitney U tests with Bonferroni corrections were used to compare individual bile acids in maternal and umbilical cord samples. Student T-tests were used to compare values from paired umbilical cord artery and vein samples. Ratios were calculated using mean values for bile acids. Statistical analysis was performed using Graph Pad Prism (Graph Pad Software Inc, CA) and R (R Foundation for Statistical Computing, Vienna, Austria).

## Results

### Analysis of maternal and fetal total serum bile acids

**Figure 1:**
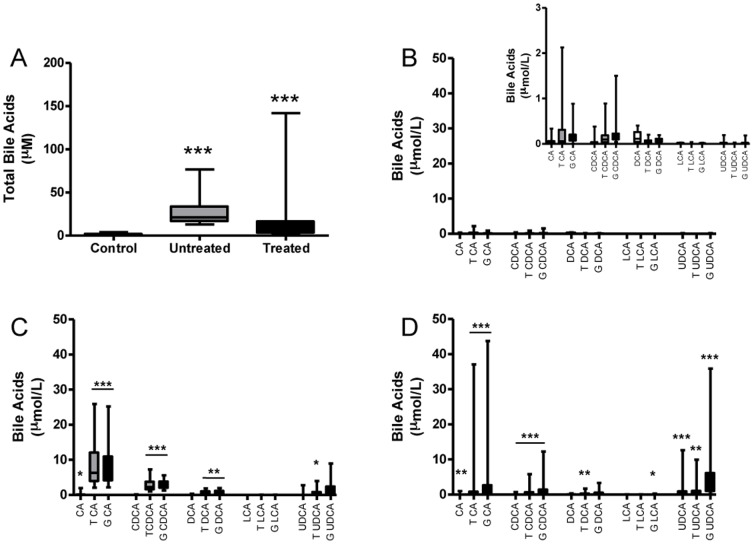
Total serum bile acids and HPLC-MS/MS analysis of bile acid profiles in maternal serum. Total serum bile acids in control, untreated ICP and UDCA-treated ICP maternal serum (A). Maternal serum bile acid profiles in normal (B), untreated ICP (C) and UDCA-treated ICP pregnancies (D). In panels B–D, the serum bile acid level is shown using equivalent Y-axes in each group. However, given the significantly lower levels of bile acids in control serum the inset panel demonstrates these data using a smaller scale. * p = <0.05, ** p = <0.001, *** p = <0.0001. Panel C comparisons vs. control, panel D comparisons vs. untreated ICP.

HPLC-MS/MS analysis of serum showed significantly higher total bile acid levels in samples from women with ICP than controls (p = <0.0001) (Figure 1A). Total serum bile acid levels were significantly lower in UDCA-treated women compared to those who were not treated (p = <0.0001).

**Figure 2:**
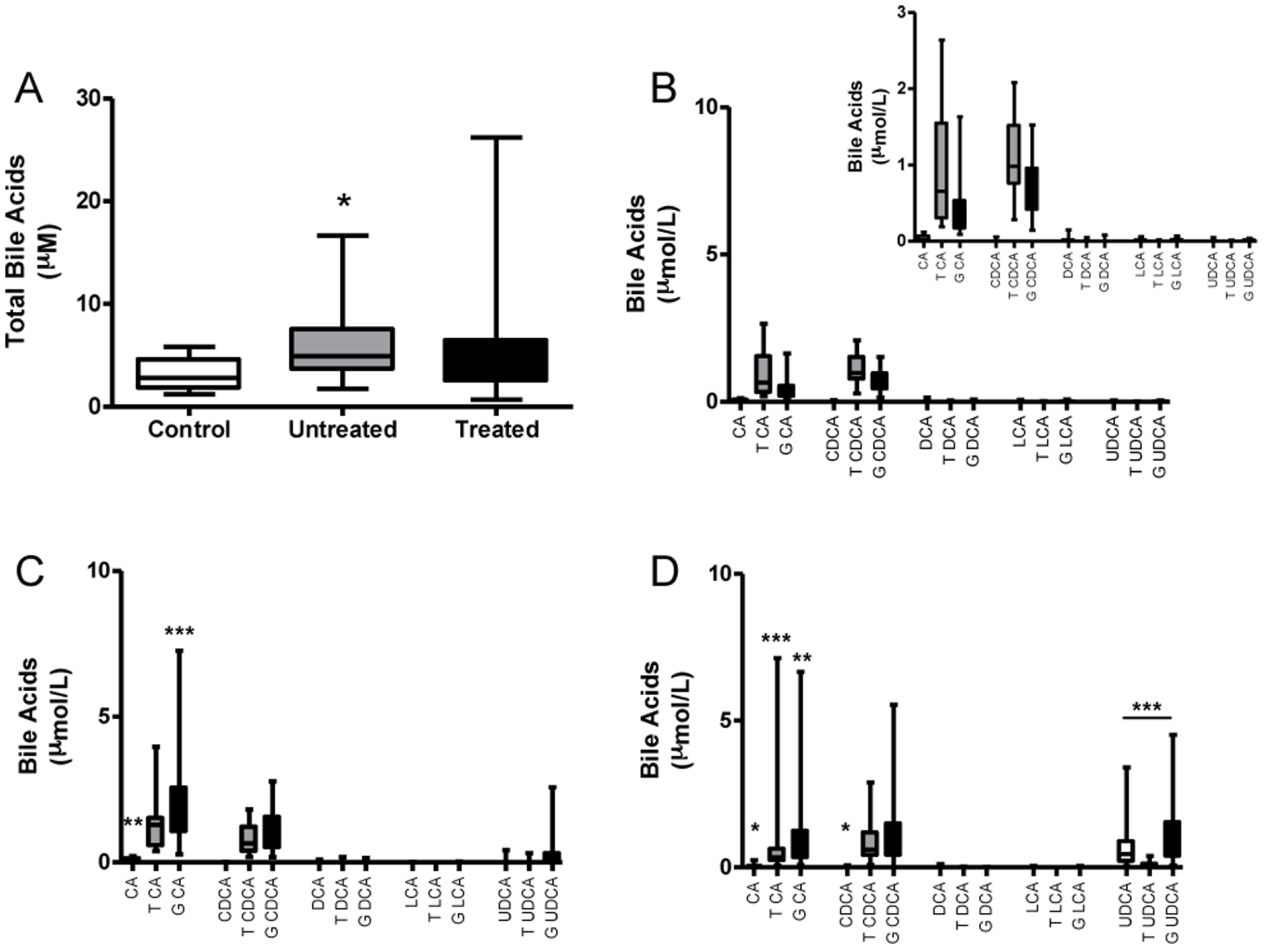
Total serum bile acids and HPLC-MS/MS analysis of bile acid profiles in umbilical cord serum. Total serum bile acids in control, untreated ICP and UDCA-treated ICP umbilical cord serum (A). Umbilical cord serum bile acid profiles in normal (B), untreated ICP (C) and UDCA-treated ICP pregnancies (D). In panels B–D, the serum bile acid level is shown using equivalent Y-axes in each group. However, given the significantly lower levels of bile acids in control serum the inset panel demonstrates these data using a smaller scale. * p = <0.05, ** p = <0.001, *** p = <0.0001. Panel C comparisons vs. control, panel D comparisons vs. untreated ICP.

Total serum bile acids were significantly higher in umbilical cord blood samples from ICP-pregnancies than in those from controls (p = <0.05). UDCA treatment reduced total bile acids in umbilical cord blood but this did not reach statistical significance (Figure 2A).

### Analysis of bile acid profiles in maternal serum and the effect of UDCA-treatment

The predominant species responsible for the elevated levels in ICP were the taurine and glycine conjugates of CA and CDCA, which were increased significantly in ICP samples compared to controls (Figure 1B and C, Table 2). Unconjugated CA and the conjugated forms of the derivative of CA, DCA, were also significantly raised in untreated ICP serum (Figure 1C, Table 2). The ratio of primary bile acids (CA: CDCA) increased from 1.00 in controls to 3.26 in untreated ICP samples. No significant change in the levels of unconjugated or conjugated LCA were observed in untreated ICP samples compared with controls.

**Table 2:** Comparisons of bile acid profiles in maternal serum.

	Median (IQR) (µmol/L)			Control vs. Untreated	Untreated vs. UDCA Treated	Controls vs. UDCA Treated
	Control	Untreated	Treated	*P* value	*P* value	*P* value
**Total bile acid**	1.20 (0.48–1.96)	21.30 (15.83–33.79)	6.70 (2.56–16.41)	<0.0001 ***	<0.0001 ***	<0.0001 ***
**Total CA**	0.25 (0.15–0.52)	14.17 (9.44–24.22)	1.01 (0.37–3.80)	<0.0001 ***	<0.0001 ***	0.0028 **
CA	0.04 (0.02–0.06)	0.12 (0.03–0.20)	0.03 (0.00–0.08)	0.0360 *	0.0084 **	0.5241
TCA	0.06 (0.02–0.31)	6.325 (3.78–12.11)	0.32 (0.11–0.87)	< 0.0001 ***	< 0.0001 ***	0.0112 *
GCA	0.12 (0.06–0.20)	5.47 (3.92–10.96)	0.62 (0.23–2.63)	< 0.0001 ***	< 0.0001 ***	0.0004 **
**Total CDCA**	0.32 (0.13–0.81)	5.49 (3.64–7.23)	1.15 (0.34–2.15)	<0.0001 ***	<0.0001 ***	0.0032 **
CDCA	0.00 (0.00–0.03)	0.02 (0.00–0.09)	0.01 (0.00–0.05)	0.3913	0.7069	0.5487
TCDCA	0.09 (0.03–0.19)	2.22 (1.38–3.78)	0.30 (0.12–0.70)	< 0.0001 ***	<0.0001 ***	0.0041 **
GCDCA	0.16 (0.08–0.23)	2.67 (1.85–3.85)	0.83 (0.24–1.44)	< 0.0001 ***	< 0.0001 ***	0.0016 **
**Total DCA**	0.27 (0.14–0.44)	0.98 (0.34–2.17)	0.36 (0.13–0.87)	0.0027 **	0.0271 *	0.1749
DCA	0.11 (0.03–0.26)	0.03 (0.0–0.16)	0.05 (0.15–0.10)	0.0574	0.3314	0.0259 *
TDCA	0.03 (0.0–0.08)	0.45 (0.16–1.04)	0.10 (0.03–0.25)	0.0002 **	0.0012 **	0.0208 *
GDCA	0.05 (0.03–0.12)	0.37 (0.14–1.15)	0.20 (0.05–0.54)	0.0013 **	0.1496	0.0109 *
**Total LCA**	0.04 (0.03–0.05)	0.04 (0.02–0.06)	0.05 (0.03–0.09)	0.9280	0.2628	0.1671
LCA	0.02 (0.01–0.03)	0.01 (0.0–0.02)	0.00 (0.00–0.02)	0.1023	1.000	0.0428 *
TLCA	0.00 (0.00–0.01)	0.00 (0.00–0.03)	0.01 (0.00–0.03)	0.6337	0.4834	0.1264
GLCA	0.02 (0.01–0.02)	0.02 (0.01–0.03)	0.03 (0.02–0.05)	0.4806	0.0462 *	0.0027 **
**Total UDCA**	0.03 (0.01–0.08)	0.07 (0.02–3.56)	3.37 (1.06–8.78)	0.1289	0.0003 ***	<0.0001 ***
UDCA	0.00 (0.00–0.03)	0.01 (0.00–0.06)	0.25 (0.08–0.92)	0.3152	< 0.0001 ***	< 0.0001 ***
TUDCA	0.00 (0.00–0.01)	0.03 (0.00–0.84)	0.45 (0.11–1.08)	0.0110 *	0.0045 **	< 0.0001 ***
GUDCA	0.01 (0.01–0.03)	0.04 (0.00–2.41)	2.10 (0.80–6.12)	0.1482	0.0004 ***	< 0.0001 ***
HDCA	0.00 (0.00–0.03)	0.00 (0.00–0.00)	0.00 (0.00–0.02)	0.0644	0.1183	0.3665
HCA	0.00 (0.00–0.01)	0.00 (0.00–0.02)	0.00 (0.00–0.00)	0.8230	0.7163	0.9723
άMCA	0.00 (0.00–0.00)	0.00 (0.00–0.00)	0.00 (0.00–0.03)	-	-	-
βMCA	0.00 (0.00–0.00)	0.00 (0.00–0.00)	0.00 (0.00–0.00)	-	-	-

CA – cholic acid, CDCA – chenodeoxycholic acid, DCA – deoxycholic acid, acid, LCA – lithocholic acid, UDCA – ursodeoxycholic acid, T and G prefixes denotes glycine and taurine conjugation respectively, − = statistical analysis not performed as bile acid levels at the limit of detection.

UDCA-treatment resulted in a significant decrease (p = <0.0001) in total serum bile acids in the maternal compartment (Figure 1A), due to significant reductions in the levels of the taurine and glycine conjugates of CA and CDCA (Figure 1D and Table 2). The levels of unconjugated LCA were significantly reduced and the levels of glycine conjugated LCA significantly increased compared controls. However the overall contribution of LCA to the bile acid pool remained low at 0.7%. The significant increases in the levels of both the unconjugated and conjugated forms of UDCA in the treated samples resulted in a decrease in the ratio of primary bile acids (CA:CDCA) from 3.26 in untreated ICP samples to 1.95 in UDCA-treated samples.

**Figure 3:**
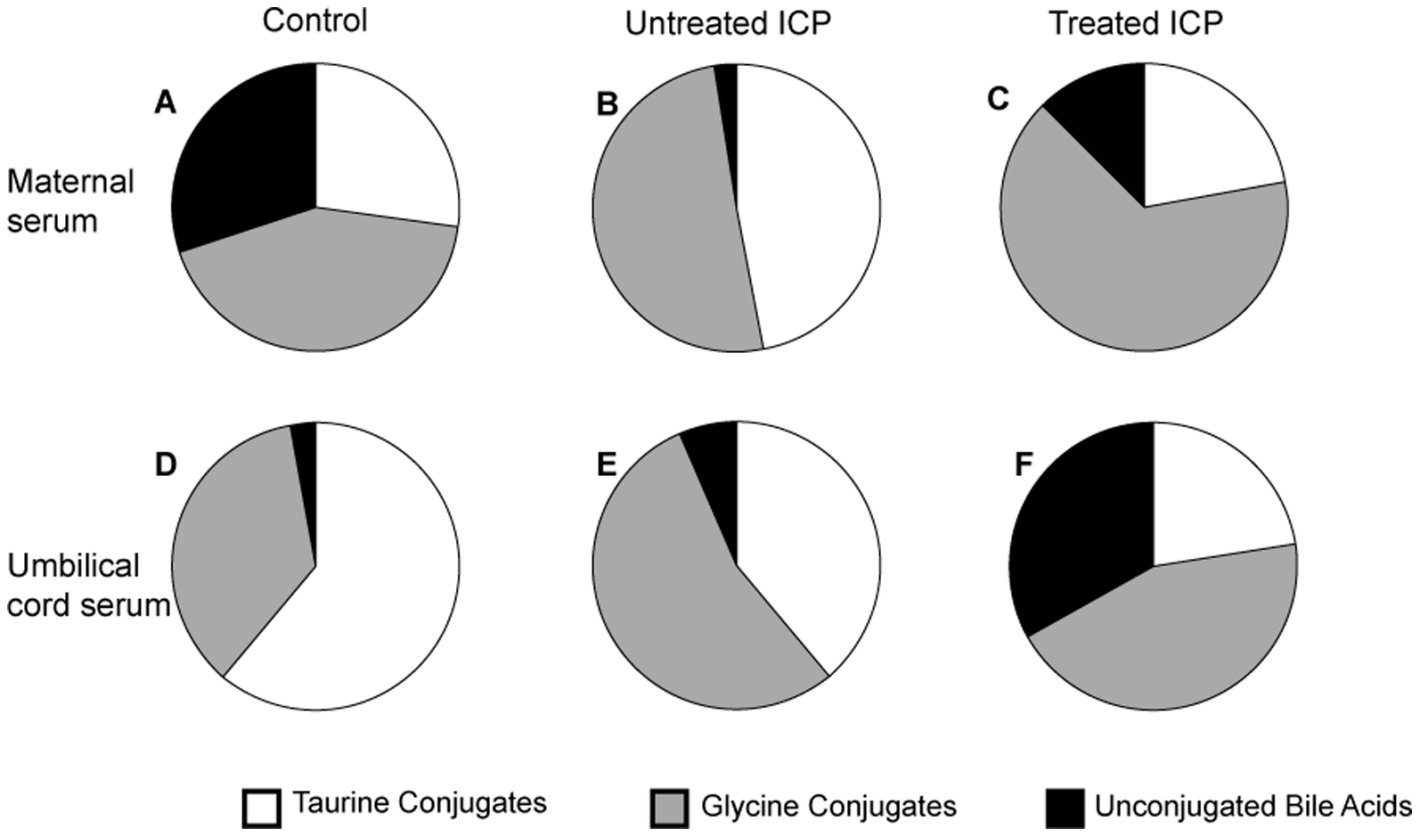
The distribution of unconjugated, taurine and glycine conjugated bile acids in maternal and cord serum. Upper panel shows data from maternal samples from normal (A), untreated ICP (B) and treated ICP (C) pregnancies and lower panel shows data from umbilical cord blood samples from normal (D), untreated ICP (E) and treated ICP (F) pregnancies.

UDCA also had a qualitative effect on the maternal bile acid pool (Figure 3). In control maternal serum the bile acid pool comprised roughly equal amounts of free, taurine and glycine conjugated bile acids. In ICP these ratios are altered due to large increases in conjugated bile acids. UDCA-treatment altered the bile acid profile with reduced proportions of unconjugated and taurine conjugated bile acids and an increased proportion of glycine conjugates, due to a significant increase in GUDCA.

### Analysis of bile acid profiles in fetal serum and the effect of UDCA-treatment

The levels of bile acids in umbilical cord artery and umbilical cord vein serum were not significantly different in untreated ICP and control samples (Table 3). In UDCA-treated cord serum samples there were significantly higher levels of unconjugated UDCA in umbilical cord vein serum. However, given that this was the only significant difference subsequent analysis of umbilical cord blood samples was undertaken on mixed samples containing blood from both the umbilical cord artery and vein.

**Table 3:** Comparisons of bile acid profiles in umbilical cord artery and umbilical cord vein samples.

	CONTROL			UNTREATED			TREATED		
	Cord Artery (µmol/L)	Cord Vein (µmol/L)	P value	Cord Artery (µmol/L)	Cord Vein (µmol/L)	P value	Cord Artery (µmol/L)	Cord Vein (µmol/L)	P value
**TCA**	0.92 (0.26)	0.93 (0.25)	0.904	1.51 (0.33)	1.58 (0.53)	0.784	0.56 (0.16)	0.58 (0.18)	0.551
**GCA**	0.47 (0.13)	0.45 (0.12)	0.707	2.92 (0.74)	2.89 (0.92)	0.961	1.21 (0.65)	1.31 (0.73)	0.320
**CA**	0.02 (0.01)	0.02 (0.01)	0.160	0.11 (0.06)	0.12 (0.02)	0.508	0.07 (0.02)	0.08 (0.02)	0.214
**TCDCA**	0.96 (0.12)	1.13 (0.20)	0.281	0.91 (0.17)	0.82 (0.18)	0.08	0.12 (0.36)	1.13 (0.34)	0.882
**GCDCA**	0.69 (0.14)	0.67 (0.12)	0.751	1.38 (0.28)	1.38 (0.28)	0.995	3.02 (2.18)	2.88 (2.01)	0.423
**CDCA**	0.003 (0.003)	0.005 (0.005)	0.339	0.01 (0.005)	0.01 (0.005)	0.518	0.03 (0.01)	0.02 (0.01)	0.539
**TDCA**	0.01 (0.006)	0.00 (0.00)	0.148	0.01 (0.01)	0.007 (0.007)	0.356	0.002 (0.002)	0.00 (0.00)	0.356
**GDCA**	0.02 (0.01)	0.0007 (0.0007)	0.201	0.02 (0.01)	0.02 (0.008)	0.747	0.004 (0.004)	0.002 (0.001)	0.646
**DCA**	0.006 (0003)	0.01 (0.01)	0.672	0.002 (0.002)	0.01 (0.005)	0.084	0.02 (0.008)	0.02 (0.005)	0.431
**TLCA**	0.001 (0.001)	0.00 (0.00)	0.339	0.001 (0.001)	0.00 (0.00)	0.356	0.004 (0.004)	0.007 (0.005)	0.243
**GLCA**	0.02 (0.006)	0.02 (0.004)	0.188	0.02 (0.001)	0.02 (0.001)	0.846	0.02 (0.002)	0.02 (0.003)	0.357
**LCA**	0.01 (0.01)	0.01 (0.005)	0.849	0.01 (0.006)	0.008 (0.002)	0.704	0.02 (0.005)	0.01 (0.004)	0.214
**TUDCA**	0.003 (0.002)	0.00 (0.00)	0.205	0.07 (0.05)	0.06 (0.05)	0.668	0.31 (0.21)	0.36 (0.26)	0.379
**GUDCA**	0.02 (0.004)	0.01 (0.003)	0.691	0.47 (0.34)	0.48 (0.38)	0.828	3.06 (2.43)	3.17 (2.53)	0.335
**UDCA**	0.008 (0.005)	0.007 (0.007)	0.930	0.06 (0.05)	0.07 (0.06)	0.913	0.85 (0.39)	0.90 (0.40)	0.048 *
**HDCA**	0.00 (0.00)	0.00 (0.00)	-	0.00 (0.00)	0.00 (0.00)	-	0.004 (0.004)	0.001 (0.001)	0.3632
**HCA**	0.001 (0.005)	0.02 (0.01)	0.1397	0.04 (0.008)	0.04 (0.01)	0.5961	0.07 (0.02)	0.07 (0.01)	0.9641
ά**MCA**	0.00 (0.00)	0.00 (0.00)	-	0.18 (0.14)	0.19 (0.16)	0.6255	1.08 (0.28)	1.07 (0.33)	0.8952
**βMCA**	0.00 (0.00)	0.00 (0.00)	-	0.00 (0.00)	0.00 (0.00)	-	0.00 (0.00)	0.00 (0.00)	-

See Table 1 for abbreviations. Values represent mean (SEM).

The predominant species contributing to the ICP-associated rise in fetal serum bile acids were TCA, GCA and GCDCA (Figure 2B and C, Table 4), although the increases in these individual bile acids were not statistically significant. The level of hyocholic acid, HCA, which is a 6ά -hydroxylation product of DCA, was significantly elevated in untreated ICP cord serum compared to control cord serum. The levels of the other less common bile acids, e.g. άMCA, which is the 6β -hydroxylation product of DCA, were low and unchanged. The ratio of the primary bile acids (CA: CDCA) in umbilical cord blood samples increased from 0.78 in controls and 1.89 in untreated ICP samples.

**Table 4:** Comparisons of bile acid profiles in fetal serum.

	Median (IQR) (µmol/L)			Control vs Untreated	Untreated vs. UDCA Treated	Controls vs UDCA Treated
	Control	Untreated	Treated	*P* value	*P* value	*P* value
**Total bile acid**	2.80 (1.73–4.62)	4.94 (3.56–7.57)	4.34 (2.44–6.50)	0.0093 **	0.3001	0.0580
**Total CA**	1.00 (0.46–1.99)	3.47 (1.47–4.18)	0.91 (0.49–2.06)	0.0011 **	0.0002 ***	0.9165
CA	0.00 (0.00–0.07)	0.07 (0.04–0.14)	0.05 (0.03–0.07)	0.0008 **	0.0171 *	0.0039 **
TCA	0.65 (0.29–1.55)	1.29 (0.55–1.53)	0.34 (0.21–0.64)	0.0829	<0.0001 ***	0.0481 *
GCA	0.34 (0.16–0.54)	1.97 (1.03–2.56)	0.54 (0.30–1.26)	<0.0001 ***	0.0011 **	0.0413 *
**Total CDCA**	1.76 (1.20–2.12)	1.91 (1.10–2.70)	1.53 (0.92–2.77)	0.7549	0.7809	0.8782
CDCA	0.00 (0.00–0.00)	0.00 (0.00–0.02)	0.02 (0.00–0.03)	0.0603	0.0334 *	0.0018 **
TCDCA	0.98 (0.75–1.52)	0.65 (0.35–1.22)	0.62 (0.38–1.20)	0.1551	0.8185	0.0490 *
GCDCA	0.54 (0.41–0.96)	1.01 (0.48–1.57)	0.83 (0.42–1.58)	0.0770	0.7543	0.1728
**Total DCA**	0.01 (0.00–0.03)	0.02 (0.00–0.04)	0.00 (0.00–0.03)	0.3299	0.0829	0.6719
DCA	0.00 (0.00–0.02)	0.01 (0.00–0.02)	0.00 (0.00–0.03)	-	-	-
TDCA	0.00 (0.00–0.01)	0.00 (0.00–0.01)	0.00 (0.00–0.00)	-	-	-
GDCA	0.00 (0.00–0.01)	0.00 (0.00–0.01)	0.00 (0.00–0.00)	-	-	-
**Total LCA**	0.02 (0.02–0.05)	0.02 (0.01–0.02)	0.02 (0.01–0.03)	0.0500 *	0.8674	0.0720
LCA	0.00 (0.00–0.02)	0.00 (0.00–0.01)	0.00 (0.00–0.01)	-	-	-
TLCA	0.00 (0.00–0.00)	0.00 (0.00–0.00)	0.00 (0.00–0.00)	-	-	-
GLCA	0.02 (0.01–0.02)	0.02 (0.01–0.02)	0.01 (0.00–0.02)	0.3486	0.8891	0.1293
**Total UDCA**	0.01 (0.01–0.03)	0.03 (0.00–0.44)	1.37 (0.60–2.60)	0.3061	<0.0001 ***	<0.0001 ***
UDCA	0.00 (0.00–0.00)	0.00 (0.00–0.03)	0.45 (0.19–0.90)	0.3734	<0.0001 ***	<0.0001 ***
TUDCA	0.00 (0.00–0.00)	0.00 (0.00–0.04)	0.06 (0.03–0.13)	0.1560	0.0004 ***	<0.0001 ***
GUDCA	0.01 (0.01–0.02)	0.03 (0.00–0.31)	0.60 (0.36–1.55)	0.2821	<0.0001 ***	<0.0001 ***
HyoDCA	0.00 (0.00–0.00)	0.00 (0.00–0.00)	0.00 (0.00–0.00)	-	-	-
HyoCA	0.00 (0.00–0.01)	0.03 (0.02–0.06)	0.06 (0.04–0.08)	0.0028 **	0.0027 **	<0.0001 ***
άMCA	0.00 (0.00–0.00)	0.04 (0.00–0.21)	0.97 (0.73–1.21)	-	<0.0001 ***	-
βMCA	0.00 (0.00–0.00)	0.00 (0.00–0.00)	0.00 (0.00–0.00)	-	-	-

See Table 1 for abbreviatons.

UDCA-treatment resulted in a non-significant decrease in the total bile acid level in cord serum (p = 0.069) (Figure 2A). Following treatment there were significant decreases in unconjugated and conjugated CA, and significant increases in unconjugated CDCA and GDCA (Figure 2D and Table 4). GUDCA was the predominant form of UDCA detected. Unconjugated and taurine conjugated LCA were at the limit of detection and therefore it was not possible to perform statistical analysis. The levels of GLCA were not significantly changed. The levels of HCA were significantly increased in UDCA-treated cord serum compared to untreated and control samples (p = 0.0027 and <0.0001, respectively). The ratio of primary bile acids (CA:CDCA) in umbilical cord blood following treatment was 0.81.

The umbilical cord blood bile acid pool in samples from control is predominantly composed of taurine conjugates (Figure 3). The taurine:glycine ratio was almost completely reversed in untreated-ICP samples. Following treatment with UDCA, the level of unconjugated bile acids was increased due to unconjugated UDCA.

### Analysis of the relationship between maternal and fetal serum bile acids

**Figure 4:**
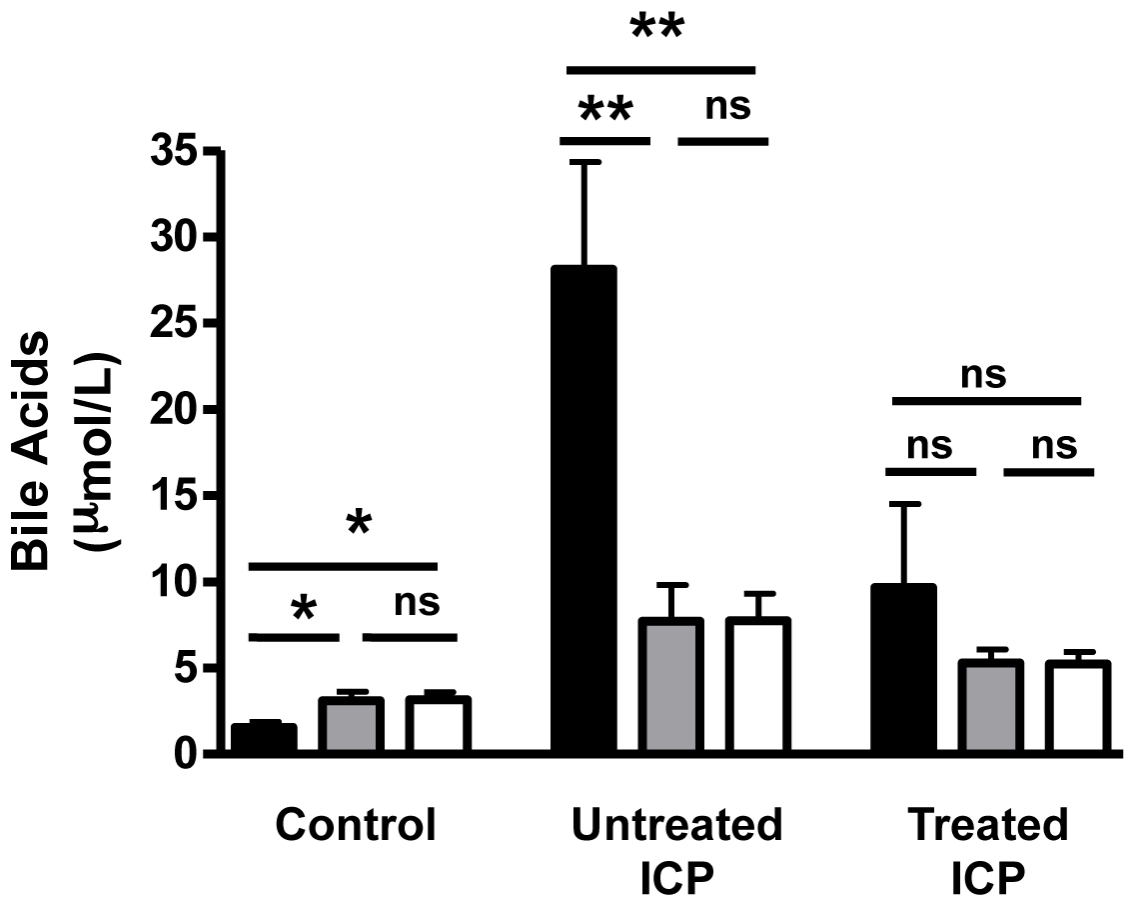
Transplacental total bile acid gradients in ICP cases and controls. Graphs representing the differences in the levels of total bile acids between maternal, umbilical cord artery and vein serum samples from normal (A) (n = 15), untreated ICP (B) (n = 7) and treated ICP (C) (n = 5) pregnancies. Black bars = maternal samples, grey bars = umbilical cord vein samples, white bars = umbilical cord artery samples. * p = <0.05, maternal total bile acid vs. cord artery total bile acid and cord vein total bile acid. ** p = <0.005, maternal total bile acid vs. cord artery total bile acid and cord vein total bile acid. ns = not significant.

In controls the umbilical cord bile acids were higher than those in the maternal serum samples (Figure 4A). In untreated ICP the level of bile acids was increased in both the maternal and fetal samples, and the transplacental gradient was reversed Figure 4B). In samples from UDCA-treated ICP there was a reduction of the steepness of the transplacental gradient (Figure 4C).

## Discussion

We have demonstrated that total serum bile acids are significantly elevated in both maternal and fetal serum from ICP pregnancies, and are reduced by UDCA-treatment. In maternal serum, elevations were predominantly due to increased conjugated primary bile acids (CA and CDCA). UDCA-treatment resulted in significantly higher levels of unconjugated UDCA in umbilical cord vein samples compared with umbilical cord artery samples. No other differences between the levels of bile acids in the umbilical cord artery and vein were observed. ICP was associated with a reversal of the transplacental gradient of bile acids and UDCA treatment reduced this gradient.

No significant increase was observed in the level of any of unconjugated or taurine conjugated LCA, in samples collected from women with ICP. Although there was significantly more glycine conjugated LCA in samples from ICP women treated with UDCA compared to controls, it should be noted that the proportion of total LCA in these samples remained less than 1% (<0.1 µmol/L), and this change is therefore unlikely to be of clinical significance. LCA is a monohydroxy derivative of CDCA and is more hydrophobic and toxic than other bile acids. Of note, one group reported extraordinarily high serum levels of LCA in ICP [27]. However, their data lack confirmation. Here we present comprehensive bile acid profiles that were measured using state-of-the-art technology [28], [29].

UDCA treatment reduced the total serum bile acid level in both the maternal and fetal compartments and also had an effect on the profiles of bile acids in both compartments, which is of clinical relevance as bile acids other than UDCA are considered cytotoxic. It is noteworthy that the influence of UDCA on the bile acid composition in maternal serum was to restore the pool to be similar to that in the control samples, whilst in the fetal samples, the difference between control and ICP serum remained considerable, with a preponderance of unconjugated bile acids. Whether these changes are beneficial to either the mother or fetus in terms of clinical outcomes remains to be seen in a large scale randomised controlled trial. However evidence from case reports, small trials and a meta-analysis indicate that UDCA has a beneficial effect for the mother in improving serum biochemical abnormalities and symptoms, consistent with the changes that are reported in this study. Furthermore, it may also protect the fetus and therefore the ongoing alteration in the bile acid profile of the umbilical cord serum following UDCA treatment reported here is intriguing [1], [21], [22]. Not all ICP cases reported here had a biochemical improvement to treatment with UDCA. This may be explained by the genetic background of the individuals, as genetic variation in *ABCB4* has been shown to be associated with a higher rate of response to UDCA.

The magnitude of reduction for total serum bile acids in umbilical cord blood following UDCA treatment is not as great as in previously published studies [12], [13]. This may be due to different durations of treatment or patient compliance with treatment. Alternatively, it may reflect the severity of maternal cholestasis as the cases reported in this study had less marked hypercholanaemia than the mothers in other studies.

The transplacental gradients for total bile acids were similar to those previously published [10]–[13]. In normal pregnancy this gradient is in the direction of fetus to mother, thereby aiding elimination of these toxic compounds from the fetal compartment. In untreated ICP, the transplacental gradient is reversed and therefore bile acids have the potential to accumulate in the fetal compartment. However, given that the level of bile acids in the fetal compartment remain relatively low in comparison to the maternal level, the data presented here support the proposal that there is active transport of bile acids across the placenta in order to protect the fetus from the potentially deleterious effects of high levels of bile acids. Several studies have aimed to identify the transporter proteins responsible for the bile acid transfer from the maternal to the fetal compartment. The predominant biliary bile acid transporters NTCP and BSEP are not expressed in the placenta [6]–[8]. Alternative transporters that may mediate transplacental bile acid transport include BCRP, OST ά and β and the OATPs [7], [8], [30], [31]. The effect of UDCA on the transplacental gradient was less marked in this study than in *in vitro* models [32] and may reflect the heterogeneity of the group of women who were treated with regard to severity of disease and duration of treatment. However, UDCA treatment clearly improves the ICP associated reversal of the feto-maternal bile acid gradient.

In conclusion we have examined the relationship between maternal and umbilical cord serum bile acids in a large cohort of women with ICP. We have characterised the maternal and fetal bile acid pools in normal, and ICP pregnancy, and documented the effects of UDCA treatment on these. Furthermore, we have demonstrated for the first time that there are no major differences between the levels of individual bile acids in umbilical cord artery and vein serum samples. This suggests that there is no significant fetal metabolism of the raised bile acids of maternal origin in ICP. Importantly this study has not shown any clinically significant increase in the total levels of LCA in either maternal or fetal samples, either as a direct result of ICP or following UDCA treatment.
